# A Single Lung Abscess Caused by Panton-Valentine Leukocidin-Producing Methicillin-Resistant Staphylococcus aureus

**DOI:** 10.7759/cureus.61845

**Published:** 2024-06-06

**Authors:** Cheuk Cheung Derek Leung, Yu Hong Chan, Man Ying Ho, Ming Chiu Chan, Chun Hoi Chen, Ching Man Ngai, Hiu Ching Christy Chan, Yiu Cheong Yeung

**Affiliations:** 1 Medicine and Geriatrics, Princess Margaret Hospital, New Territories, HKG

**Keywords:** severe community-acquired pneumonia, necrotizing pneumonia, • lung abscess, methicillin resistant staphylococcus aureus (mrsa), panton-valentine leukocidin (pvl)

## Abstract

This case report presents a rare occurrence of a single lung abscess caused by Panton-Valentine leukocidin (PVL)-producing methicillin-resistant *Staphylococcus aureus* (MRSA) in a 38-year-old immunocompetent man. The patient, of Southeast Asian origin, presented with symptoms of fever, chest pain, cough, and shortness of breath following a recent flu-like illness. Imaging indicated a cavitary lung lesion in the left lower lobe, suggestive of a lung abscess. Initial antibiotic treatment failed, and drainage of the abscess confirmed MRSA with the PVL gene, indicating a community-acquired MRSA infection. The patient received intravenous vancomycin followed by oral linezolid, leading to the resolution of the abscess. Contact tracing and decolonization measures were implemented. This case highlights the importance of considering PVL-producing *S. aureus* as a potential pathogen in severe necrotizing pneumonia or sepsis and underscores the need for prompt diagnosis, appropriate antibiotic therapy, and infection control measures in managing such infections.

## Introduction

Methicillin-resistant *Staphylococcus aureus* (MRSA) is a strain of *S. aureus* that has developed resistance to beta-lactam antibiotics [1]. Its infections primarily affect hospitalized or residential care patients but can also impact community-dwelling individuals without recent medical procedures, known as community-acquired MRSA (CA-MRSA) infections [2]. CA-MRSA strains, in contrast to hospital-acquired MRSA (HA-MRSA), typically possess staphylococcal cassette chromosome mec type IV, generate Panton-Valentine leukocidin (PVL), and are more likely to exhibit sensitivity to various non-beta-lactam antibiotics such as co-trimoxazole, ciprofloxacin, and erythromycin [3]. It most commonly causes skin and soft tissue infections, with rare occurrences of severe and life-threatening conditions like meningitis and severe sepsis [4]. Necrotizing pneumonia has been reported in various literature in young and immunocompetent patients [4-8], often leading to the formation of multiple lung abscesses [5,8], a well-recognized continuum with necrotizing pneumonia. However, a single lung abscess confirmed to be caused by PVL-producing *S. aureus* has only been reported in a handful of case reports [9]. In this article, we report a case of a single lung abscess caused by PVL-producing MRSA in a 38-year-old immunocompetent man.

## Case presentation

A 38-year-old man of Southeast Asian origin presented in January 2023 with fever, left pleuritic chest pain, productive cough, and shortness of breath for three days. The onset of the illness was preceded by an episode of influenza-like illness two weeks prior. He worked as a construction site manual laborer with chronic smoking and alcohol use. He had a past medical history of asthma, which was well controlled without regular inhaler use, and an episode of culture-negative right upper lobe lung abscess treated with intravenous co-amoxiclav and ceftriaxone for four weeks in 2021. Neither the patient nor his close family members reported a history of significant skin infections.

**Figure 1:**
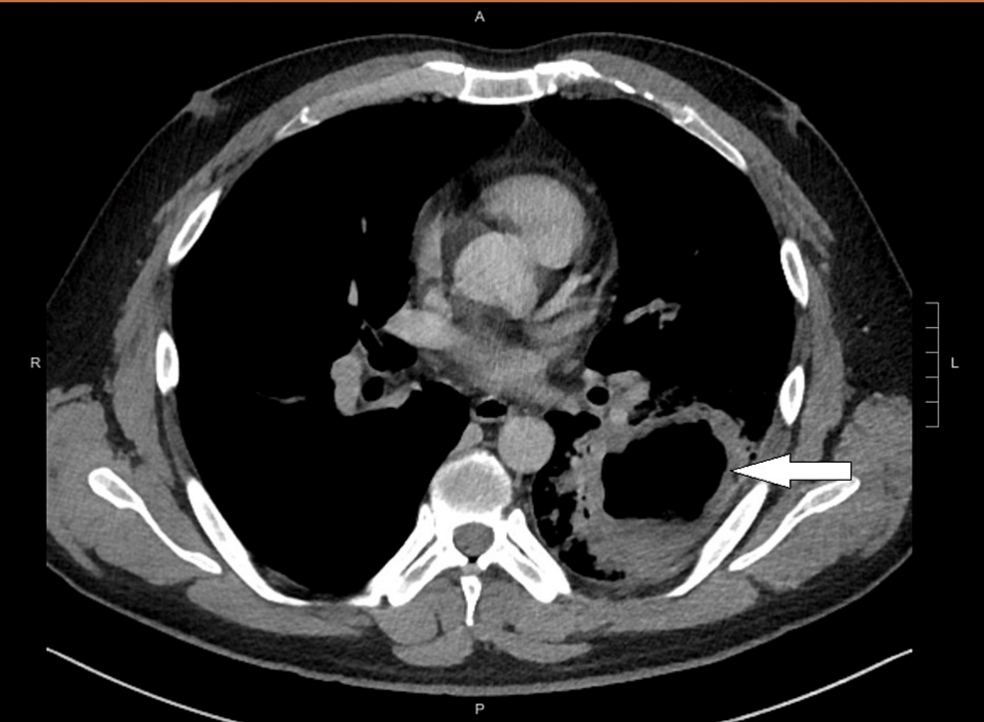
Contrast-enhanced CT image of the patient before the CA-MRSA treatment The white arrow points toward an 8.3 × 6.2 × 8.7 cm lung abscess in the left lower lobe. CA-MRSA, community-acquired methicillin-resistant *Staphylococcus aureus*

On admission, his temperature was 38.5 °C, his blood pressure was 124/75 mmHg, his pulse rate was 99 beats per minute, and his oxygen saturation was 95% on room air. The chest examination revealed coarse crackles over the left middle zone. He had an elevated white cell count of 18.7 × 109/L, C-reactive protein >320 mg/L, and procalcitonin of 2.38 ng/mL, indicating the presence of a bacterial infection. Anti-HIV antibodies were negative, and hemoglobin A1c (HbA1c) levels were normal. There was no evidence of immunodeficiency. A chest X-ray showed a left middle zone cavity, and a contrast CT of the thorax four days after admission showed a thick-walled cavitary lung lesion measuring 8.3 × 6.2 × 8.7 cm at the left lower lobe with an air-fluid level suggestive of a lung abscess, associated with surrounding consolidative changes, ground glass opacities, prominent mediastinal lymph nodes, and a small left pleural effusion (Figure 1).

Fibrosis was seen in the right upper lobe, where the previous lung abscess was located in 2021, with no right-sided pulmonary consolidation or discrete lung mass.

Empirical intravenous antibiotics, including metronidazole and ceftriaxone, were initiated on admission, but the patient remained febrile with no evidence of clinical improvement. Therefore, the abscess was drained, which yielded frank pus. The pus and sputum cultured with MRSA with a polymerase chain reaction test confirmed the presence of the PVL gene, signifying a CA-MRSA infection. It was resistant to cloxacillin and erythromycin but sensitive to vancomycin, sodium fusidate, rifampicin, and co-trimoxazole. Tuberculosis was ruled out with a negative acid-fast bacilli smear and a culture of pus and sputum.

**Figure 2:**
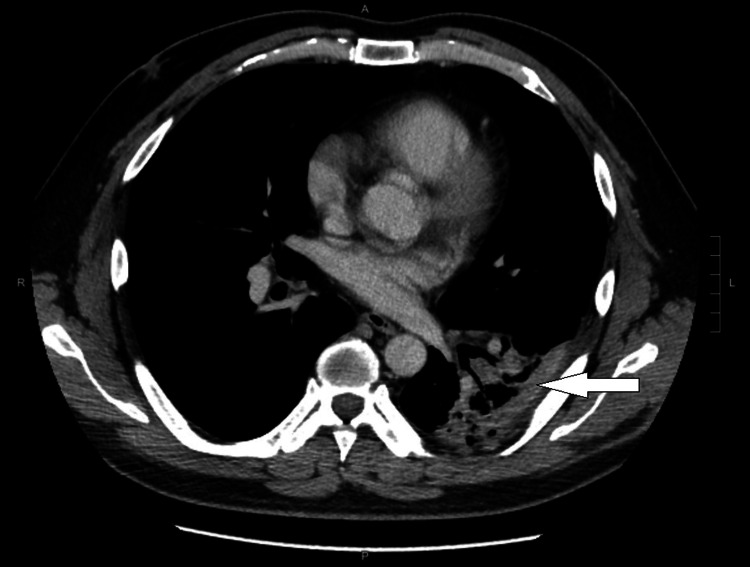
Contrast-enhanced CT image of the patient during the CA-MRSA treatment The white arrow points toward a resolving lung abscess in the left lower lobe. CA-MRSA, community-acquired methicillin-resistant *Staphylococcus aureus*

After diagnosing a PVL-producing *S. aureus* lung abscess, the Department of Health of Hong Kong was notified for contact tracing and decolonization. Antibiotics were switched to intravenous vancomycin for a total of four weeks, with a follow-up contrast CT thorax in March 2023 showing resolution of the left lower lobe lung abscess at the end of the vancomycin treatment (Figure 2).

**Figure 3:**
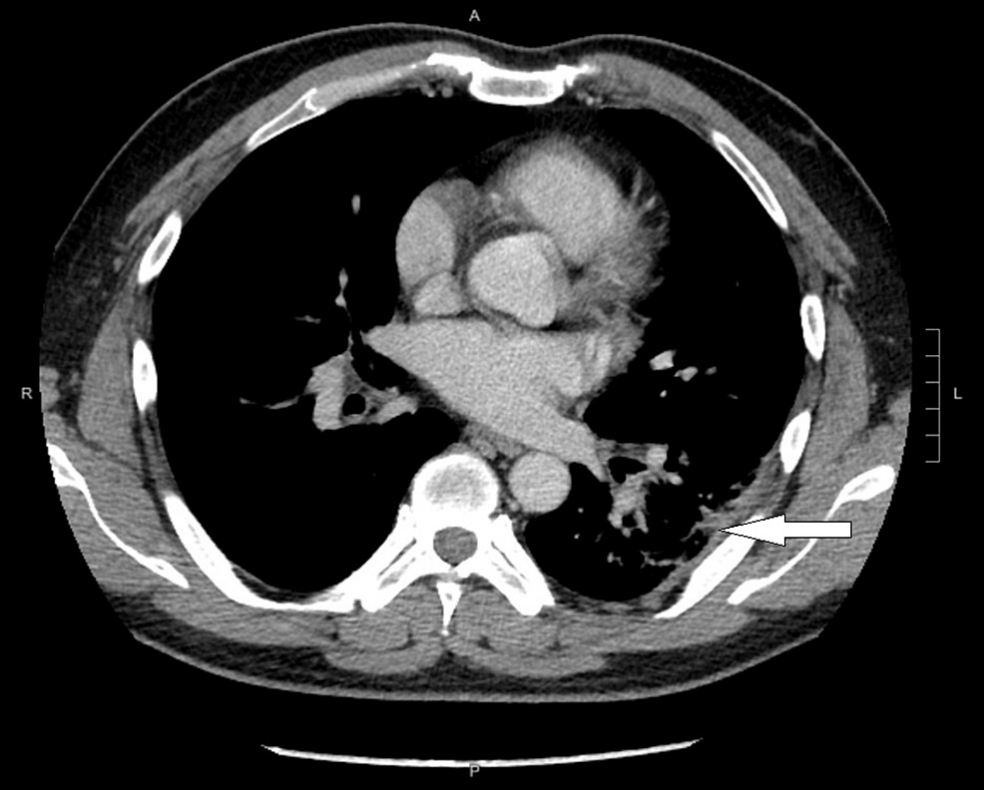
Contrast-enhanced CT image of the patient after the CA-MRSA treatment The white arrow points toward a resolved lung abscess in the left lower lobe. CA-MRSA, community-acquired methicillin-resistant *Staphylococcus aureus*

The patient recovered well, was discharged with a further two-week course of oral linezolid, and received MRSA decolonization therapy upon completion of antibiotic treatment. The follow-up contrast CT thorax in May 2023 was taken one month after the completion of oral linezolid. It showed a resolved left lower lobe abscess with residual consolidative changes and ground glass opacities (Figure 3).

## Discussion

The most common cause of lung abscess is aspiration of oral secretions by patients who have reduced consciousness. Other less common etiologies include endobronchial obstructions and hematogenous seeding of the lungs. The patient’s blood cultures were negative, and no endobronchial obstructions were evident on the first contrast CT thorax (Figure 1). Therefore, it is believed that our patient with alcohol use disorder may have aspirated while intoxicated with ethanol, resulting in a lung abscess that was subsequently infected with PVL-producing *S. aureus*. In 2021, he experienced an episode of a right upper lobe lung abscess, for which he received treatment using co-amoxiclav and ceftriaxone. These antibiotics are ineffective against PVL-producing *S. aureus*, so it was unlikely that the lung abscess in 2021 was caused by PVL-producing *S. aureus*.

CA-MRSA, or PVL-producing *S. aureus*, is a statutory notifiable disease required by law to be reported to the Department of Health in Hong Kong. A total of 640 cases were reported in 2023. PVL-producing *S. aureus *infections are primarily transmitted through direct contact with wounds, discharge, and contaminated areas [10]. It is more infectious in comparison to HA-MRSA. Therefore, if not contraindicated, all patients infected with PVL-producing *S. aureus* should undergo decolonization therapy with 4% chlorhexidine gluconate liquid soap to the scalp, hair, and whole body and 10% povidone-iodine ointment to the nostrils for five days [10].

## Conclusions

We reported a rare case of a single lung abscess confirmed to be caused by PVL-producing* S. aureus* in an immunocompetent 38-year-old alcoholic. The patient underwent treatment with drainage of the lung abscess and intravenous vancomycin, followed by oral linezolid, and made a full recovery. Contact tracing and decolonization measures were implemented. The report highlights the uncommon occurrence of PVL-producing *S. aureus *lung abscess and emphasizes the importance of decolonization therapy for these infections. It also serves as a reminder to physicians to consider PVL-producing *S. aureus *as the culprit in young patients with severe necrotizing pneumonia or sepsis.
